# Metabolomic analysis indicated changes in triacylglycerols’ levels as a result of training in Whippet dogs

**DOI:** 10.1038/s41598-023-45546-w

**Published:** 2023-10-25

**Authors:** Katarzyna Miazga, Klaudia Kopczyńska, Olga Szaluś-Jordanow, Agata Moroz-Fik, Jacek Wilczak, Karolina Barszcz, Anna Cywińska

**Affiliations:** 1 Department of Pathology and Veterinary Diagnostics, Institute of Veterinary Medicine, Warsaw University of Life Sciences-SGGW, Nowoursynowska 159C, 02-776 Warsaw, Poland; 2 Municipal Zoological Garden in Warsaw, Ratuszowa 1/3, 03-461 Warsaw, Poland; 3 Department of Functional and Organic Food, Institute of Human Nutrition Sciences, Warsaw University of Life Sciences, 02-776 Warsaw, Poland; 4 Department of Small Animal Diseases with Clinic, Institute of Veterinary Medicine, Warsaw University of Life Sciences-SGGW, Nowoursynowska 159C, 02-776 Warsaw, Poland; 5 Division of Veterinary Epidemiology and Economics, Institute of Veterinary Medicine, Warsaw University of Life Sciences-SGGW, Nowoursynowska 159C, 02-776 Warsaw, Poland; 6 Department of Physiology, Institute of Veterinary Medicine, Warsaw University of Life Sciences-SGGW, Nowoursynowska 159C, 02-776 Warsaw, Poland; 7 Department of Morphological Sciences, Warsaw University of Life Sciences, Nowoursynowska 159C, 02-776 Warsaw, Poland; 8 Faculty of Biological and Veterinary Sciences, Nicolaus Copernicus University in Torun, Lwowska 1, 87-100, Torun, Poland

**Keywords:** Metabolism, Biochemical reaction networks

## Abstract

Regular physical effort produces metabolic changes manifested as adaptation to exercise and increasing performance. In humans these changes have been characterized at metabolome level as depending on the discipline. However, all sports involve some level of changes in protein, carbohydrate and lipid metabolism. Recently, also performance horses have been subjected to metabolic analyses, but similar studies were lacking in sports dogs. In this study we performed the metabolomic analysis in plasma of Whippet dogs regularly trained and competing in coursing events, and untrained dogs of the same breed, fed with the same diet. We have also compared the hematological and blood biochemical results in these two groups of dogs. Basic blood tests indicated that enzymes related to lipid metabolism (lipase and gamma-glutamyltransferase) differed considerably between the groups. Metabolomic analysis of plasma confirmed the metabolic shift expressed as the differences in triacylglycerols levels between training and non-training dogs, aimed at improving the use of fatty acids as a source of energy during exertion. Surprisingly, other classes of metabolites were only hardly changed when comparing training and non-training Whippets.

## Introduction

Physical effort causes a number of changes in the body aimed at providing the necessary energy and maintaining homeostasis at the same time. Metabolomics is a field of science that allows to track and monitor these changes in more accurate way than previously used research methods. Large variety of different metabolites can be monitored, which is very valuable when focusing on interactions during exercise or nutritional interventions^1^. Metabolites are defined as low molecular weight (< 1500 Daltons) chemical substrates, intermediates, or end products of enzyme-mediated reactions^2^.

Metabolomics is a quickly growing field in veterinary medicine. In dogs, a large part of the published research focuses on variability of the metabolome in different disease entities and the search for metabolites that could be markers for these diseases^3–6^. Several canine diseases have been evaluated with some promise for potential biomarker and/or disease mechanism discovery^5^. It has also been proven in humans that undertaking various types of physical activity produces changes in metabolic profiles. In the case of human athletes, it has been confirmed that different types of exercise affect the metabolic pathways of compounds from various groups, including lipids, amino acids, and carbohydrates^1,7^. A word “sportomics” was created and it means the application of metabolomics in sports to investigate the metabolic effects of physical exercises on individuals, regardless of whether they are professional athletes or not^8^. Several metabolomic studies in humans have been published in this field^7,9–11^, but according to the author’s knowledge, no such research in dogs have been published.

Certain canine breeds practice sports of various disciplines. The oldest and most traditional canine discipline is racing dedicated for hounds, and currently a wide variety of race types have been created for certain breeds among hounds. Lure-coursing has been specially created for sighthounds, mainly Whippets to test their congenital chasing and hunting skills. Dogs chase a mechanized plastic lure in a grassy and open area. In contrast to the typical greyhound track racing, here the route is variable and consists of both straight lines and curves.

Numerous scientific reports confirm that in the body of dogs subjected to exercise, noticeable changes take place, characteristic for a specific type of exercise. Biochemical analysis of sighthounds’ blood after exercise, regardless of whether they chased a live prey or artificial lure, presented changes in the lactate (LA) level typical for anaerobic exercise in humans and horses^12^. It has also been reported that basic hematology parameters—red blood cells count (RBC), hematocrit (HCT) and hemoglobin concentration (HGB), are higher in racing Greyhounds compared to non-runners of other breeds^13^. Moreover, it has been proved that erythrogram parameters increase after exercise in Whippet dogs^14^. Knowing these outcomes, we decided to check if exercise-related changes are reflected by metabolomic changes in Whippets practicing lure-coursing. Thus, in the current study we investigated changes in metabolome of Whippet dogs trained for lure-coursing, compared to the same breed dogs that do not practice any sport training. Additionally, we compared basic hematological and blood biochemical profiles to check if routinely used reference values are accurate also for Whippets practicing regular sport training.

## Results

The study groups of training and non-training dogs (Table 1) did not differ significantly in terms of age and body weight.

**Table 1:** Study population.

	Training dogs	Non-training dogs	*p*
Number of animals	n = 16	n = 9	–
Sex	14 males (4 of them neutered), 2 females	4 males, 5 females	–
Age	3 (2–3.5) years	1.5 (1.5–3.6) years	0.559
Body weight	14.25 (13–14) kg	14.0 (12–14.5) kg	0.276

Age and body weight are given as medians (IQR range).

### Hematology and blood biochemistry

Most hematological and biochemical parameters remained within normal ranges for canine species recommended by the laboratory. In 5 training dogs WBC values were slightly below the reference range, but no other abnormalities were detected, so it was treated as normal for these individuals. Alike, due to these values the median value for WBC was significantly lower in training dogs, however, fold change (FC_median_) was below 1.5, so it was not interpreted as important. There were no significant differences in other hematological parameters between the groups (Table 2, Supplementary Table S1). The levels of several biochemical parameters: TP, ALB, GGT, CK, lipase, fructosamine differed significantly, being still within reference values, however, FC_median_ values were above 1.5 only for GGT and lipase (Table 3, Supplementary Table S2).

**Table 2:** Basic hematology results of training and non-training Whippets at rest.

Blood parameter	Reference interval	Training (n = 16)		Non-training (n = 9)		Training vs non-training *p*-value*	FC
		Median (IQR)	Range	Median (IQR)	Range		
WBC (G/l)	6.0–12.0	5.7 (4.75–6)	(3.7–7.4)	6.4 (6.2–7.3)	(5.1–8.6)	0.002	1.123
RBC (T/l)	5.5–8.0	7.68 (7.4–8.0)	(5.0–9.1)	7.47 (7.3–7.6)	(6.3–7.9)	0.152	− 1.028
HGB (mmol/l)	7.45–11.17	11.8 (11.1–12.4)	(7.4–13.8)	11.4 (11.2–12)	(10.1–12.5)	0.579	− 1.035
HCT (l/l)	0.37–0.55	0.55 (0.52–0.58)	(0.37–0.63)	0.53 (0.52–0.56)	(0.47–0.59)	0.579	− 1.038

*Significant at α = 0.05.

WBC—white blood cells, RBC—red blood cells, HGB—hemoglobin, HCT—hematocrit, FC—fold change.

**Table 3:** Selected biochemistry results of training and non-training Whippets at rest.

Blood parameter	Reference interval	Training (n = 16)		Non-training (n = 9)		Training versus non-training *p*-value*	FC
		Median (IQR)	Range	Median (IQR)	Range		
GGT (U/l)	5–25	8 (6–10.5)	(5–22)	5 (5–5.5)	(5–6)	0.006	1.6
CK (U/l)	5–467	135.5 (127.25–140.25)	(103–174)	99 (72–100)	(70–108)	0.003	1.369
TRI (mg/dl)	17.7–115.1	32 (27.5–44.25)	(20–59)	37 (33–40)	(29–41)	0.621	− 1.156
Lipase (U/l)	0–135	32.5 (24–48.5)	(18–134)	11 (10–14)	(11–16)	0.001	2.955
TP (g/l)	55–75	56 (55.5–58)	(52–58)	59.5 (57–62.25)	(57–63)	0.003	− 1.063
ALB (g/l)	29–43	32 (31.5–33)	(30–34)	36.5 (35.75–37)	(24–38)	0.001	− 1.16
Fructosamine (µmol/l)	225–365	331.27 ± 26.99	(288–366)	250.80 ± 29.02	(225–289)	0.004	1.32

*Significant at α = 0.05.

GGT—gamma-glutamyltransferase, CK—creatine kinase, TRI—triglycerides, TP—total protein, ALB—albumins, FC—fold change.

### Metabolites and metabolism indicators

Six hundred thirty metabolites (listed in the Methods section) and 232 metabolism indicators were tested. One hundred fifty seven metabolites (29 acylcarnitines, 1 alkaloid, 6 aminoacids related, 6 bile acids, 5 biogenic amines, 3 carboxylic acids, 7 ceramides, 2 cholesterol esters, 37 diacylglycerols, 8 dihydroceramides, 5 fatty acids, 14 glycerophospholipids, 3 hormones, 1 indole derivative, 1 nucleobase related, 29 triacylglycerols) were excluded from further analyses due to being out of the limit of detection (LOD), 35 out of 232 metabolism indicators were rejected from further analyses for the same reason. In total, 670 compounds were subjected to further analyses (Supplementary Table S3a and b).

**Figure 1:**
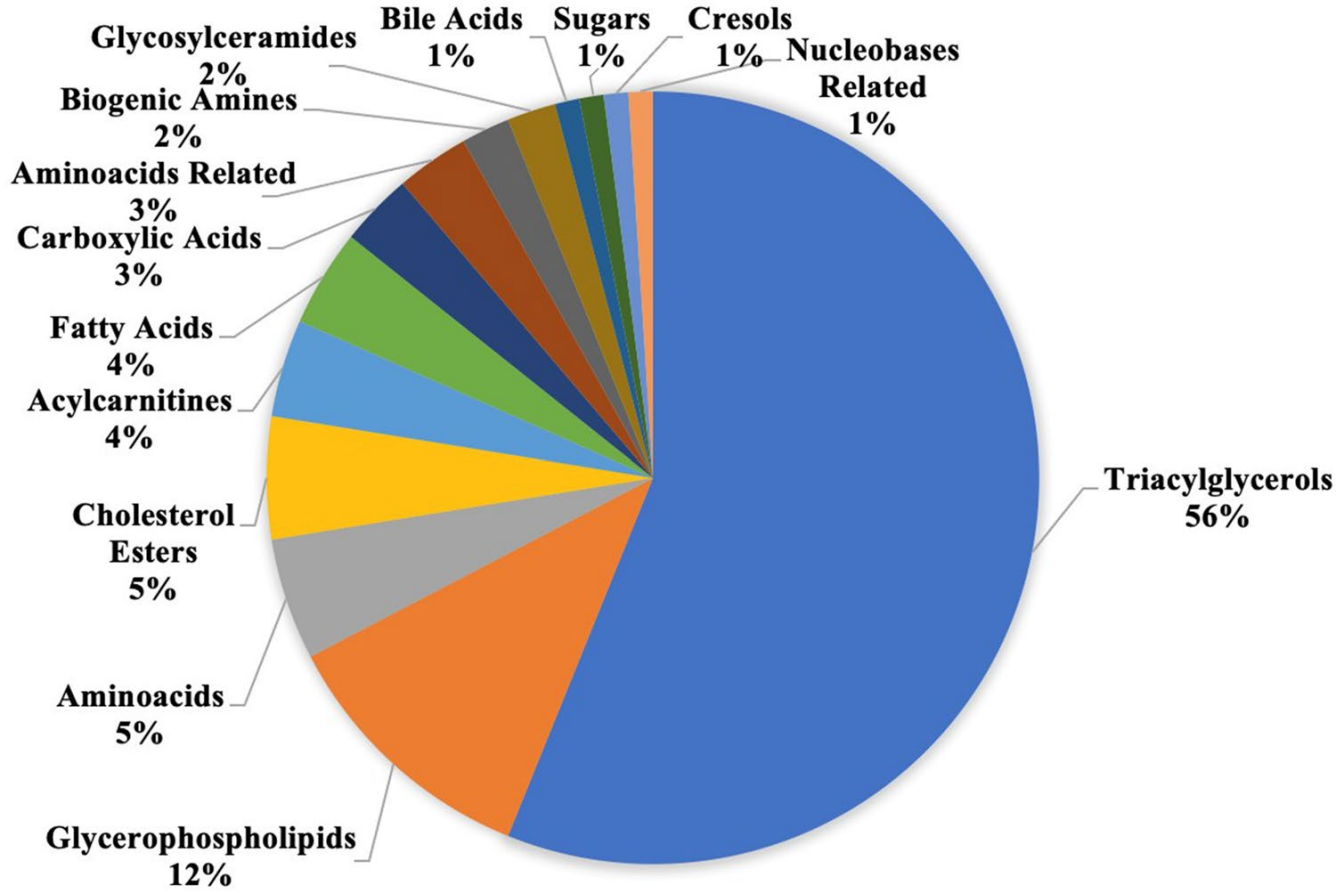
Classes of metabolites that differed significantly between training and non-training Whippets.

**Figure 2:**
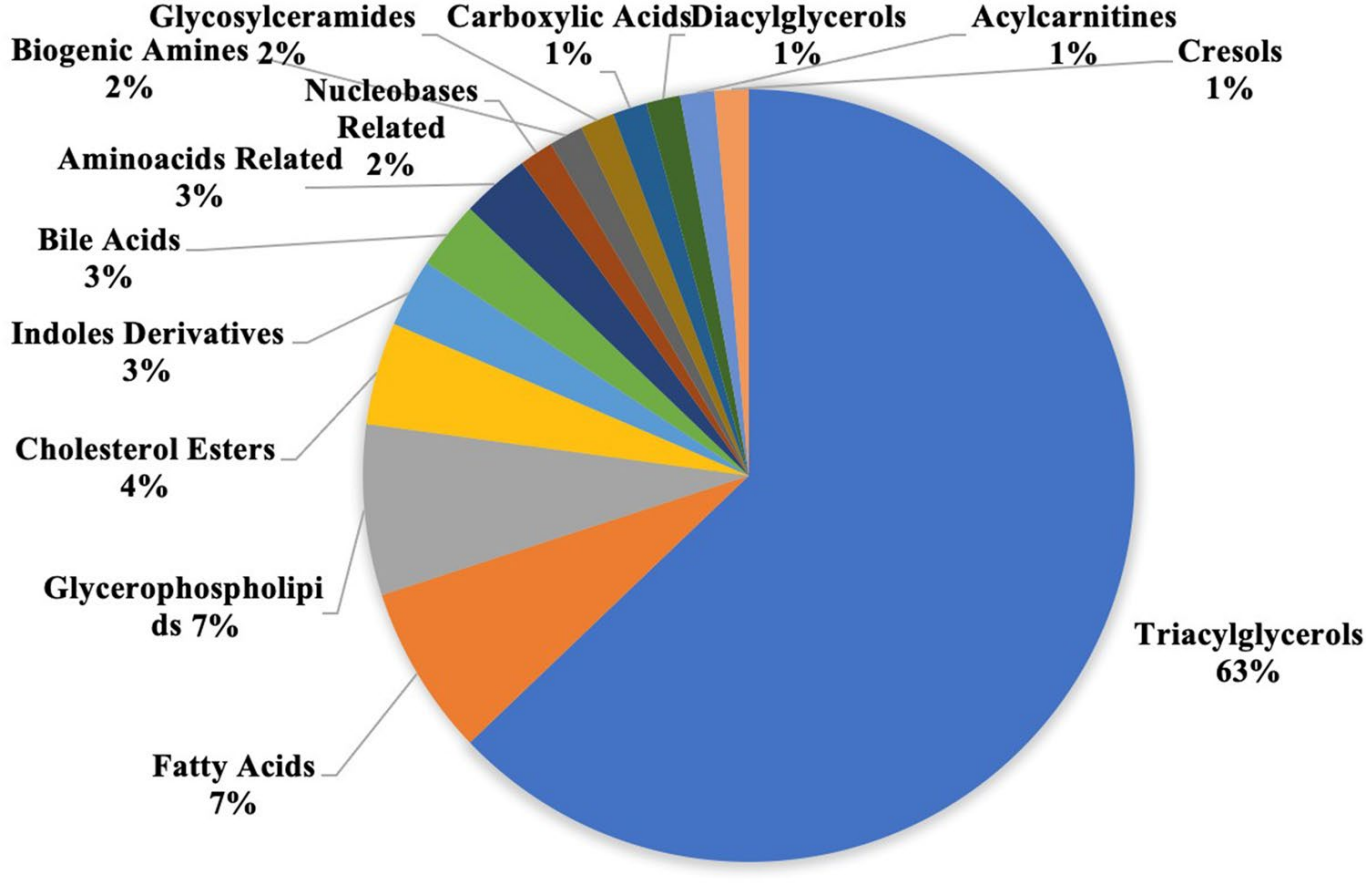
Classes of metabolites with absolute magnitude of difference between training and non-training dogs exceeding 1.5.

Ninety eight metabolites (Fig. 1) and 32 metabolism indicators differed significantly (*p* < 0.05) between groups. The criterion of absolute FC_median_ above 1.5 was fulfilled by 70 metabolites (Fig. 2) and 38 metabolism indicators, being higher or lower in the training dogs. Both criteria: significance and absolute FC_median_ above 1.5 were fulfilled by 48 metabolites (3 free fatty acids, 1 acylcarnitine, 1 aminoacids related, 1 bile acid, 1 biogenic amine, 1 carboxylic acid, 2 cholesterol esters, 1 cresol, 2 glycerophospholipids, 1 glycosylceramide, 1 nucleobases related and 33 triacylglycerols—TGs) and 18 metabolism indicators, including 7 newborn screening traits (deficiencies) designed for tests in humans. The remained 11 included: sum of betaine related metabolites, cystine synthesis, p-cresol-SO4 synthesis, sum of VLCFA-CEs, ratio of DGs to TGs, ratio of DHA to EPA, sum of MUFAs, sum of PUFAs, PLA2 activity (3), ratio of TGs to FAs, sum of purines and sum of saturated TGs (Supplementary Tables S4 and S5).

**Figure 3:**
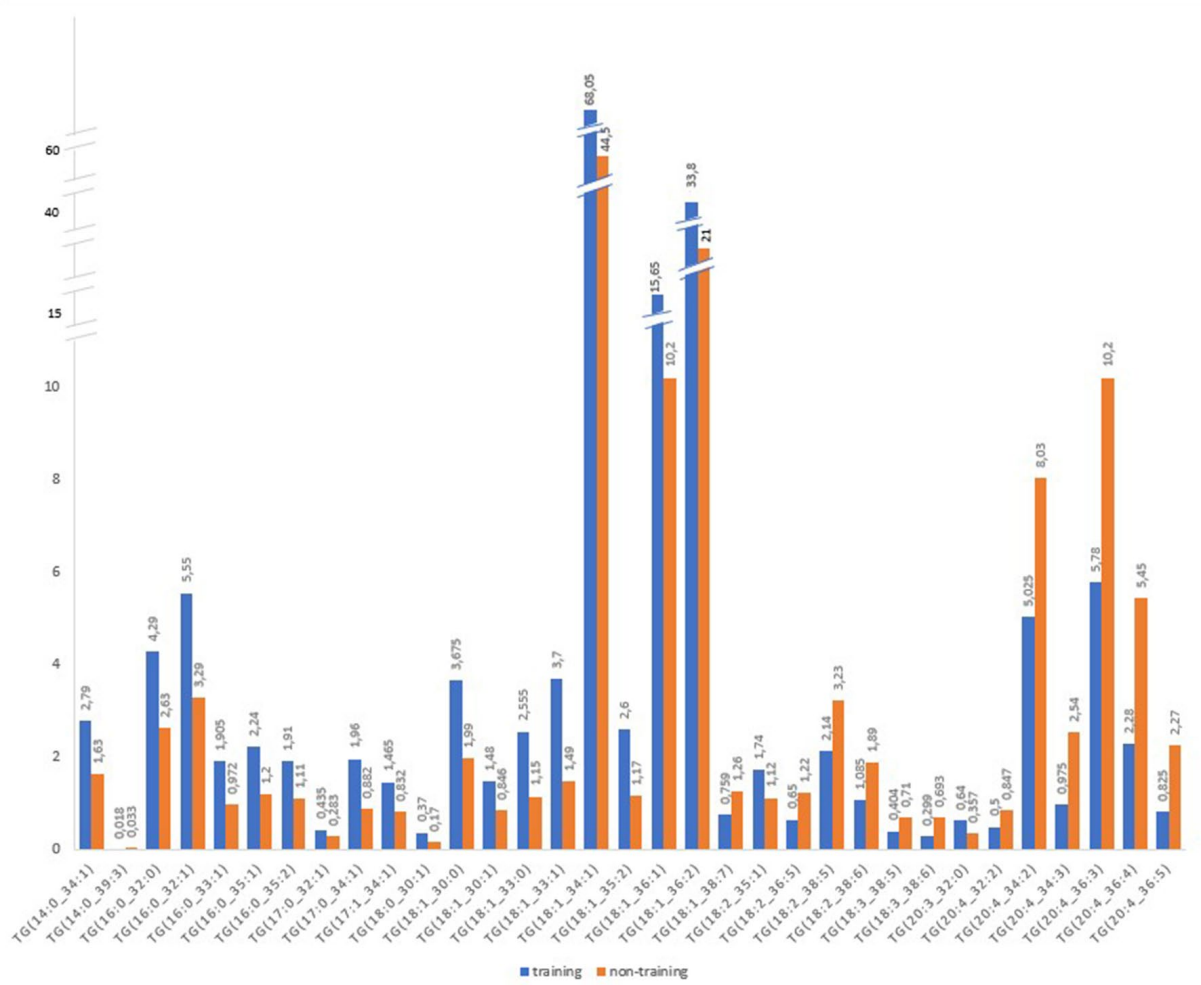
The concentrations of TGs that significantly differed between training and non-training dogs and the absolute FC_median_ was above 1.5.

Thirty three triacylglycerols (Fig. 3) that fulfilled both criteria: differed significantly between groups, and absolute FC_median_ above 1.5 contained from 14 to 20 carbon atoms residues at *sn-1* position. In the training group the concentrations of TGs containing 16 and 17 carbon residues in this position were higher than in non-training dogs. The concentrations of TGs with longer chains, containing 20 carbon atoms, except TG(20:3_32:0) were higher in the non- training group. An interesting trend has been noticed for 18 carbon residues, the concentrations of TG(18:0_30:1), TG(18:1_30:0), TG(18:1_30:1), TG(18:1_33:0), TG(18:1_33:1), TG(18:1_34:1), TG(18:1_35:2), TG(18:1_36:1), TG(18:1_36:2) and TG(18:2_35:1) were higher in training dogs, while the concentrations of TG(18:1_38:7), TG(18:2_36:5), TG(18:2_38:5), TG(18:2_38:6), TG(18:3_38:5), and TG(18:3_38:6) were higher in the non-training group. The highest concentrations (in both groups) were noted for TG(18:1_34:1) and TG(18:1_36:2), both being higher in training dogs.

**Figure 4:**
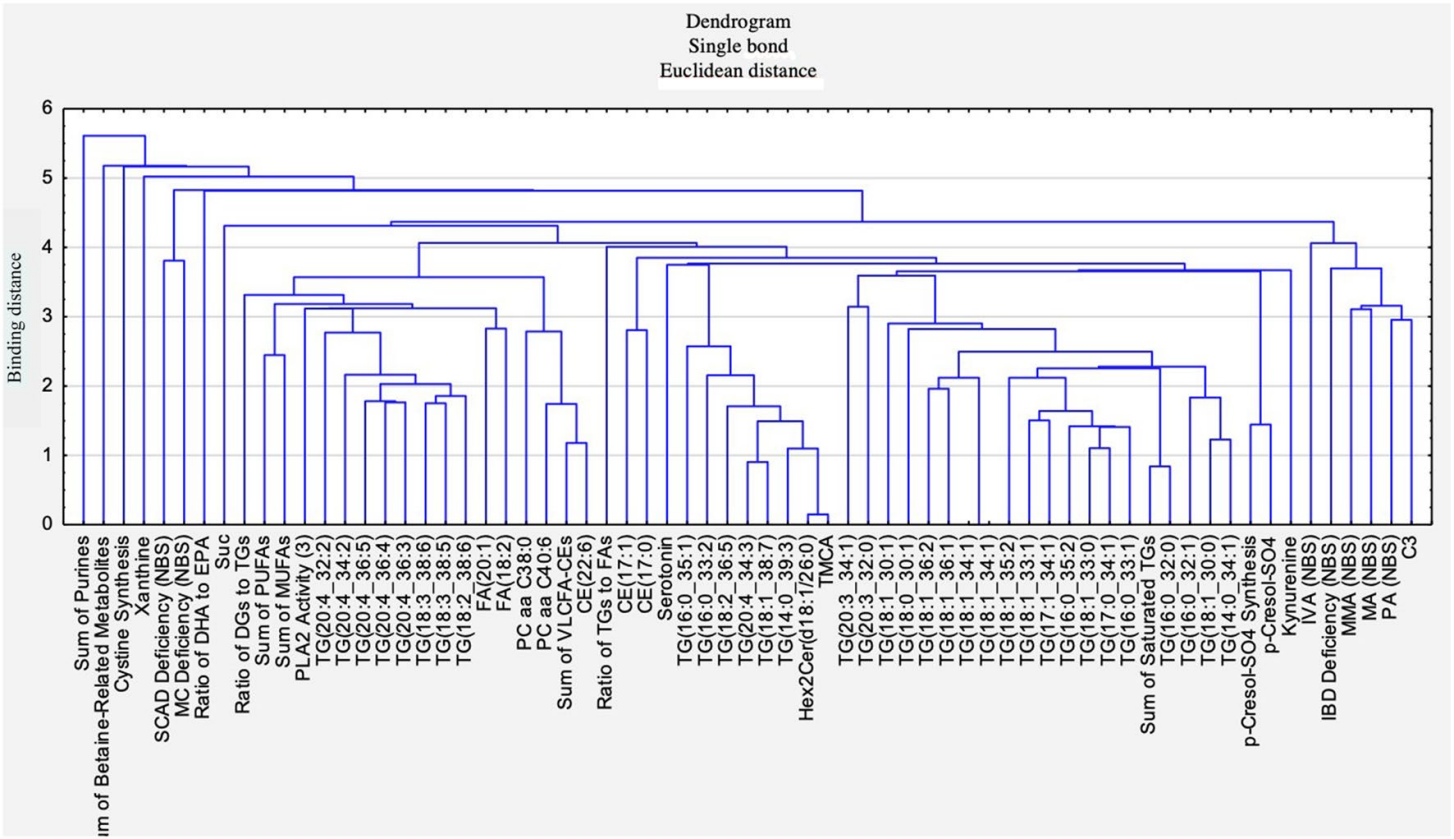
Dendrogram of the hierarchical cluster analysis of metabolites and metabolism indicators that differed significantly between training and non-training dogs and the absolute FC_median_ was above 1.5. The height of branches that link the variables represent similarities (lower located branches) and dissimilarities (higher located branches) among variables.

Hierarchical clustering of the metabolites and metabolism indicators that fulfilled both above-mentioned criteria indicated the similarities and dissimilarities among variables, as shown at the dendrogram (Fig. 4). The most similar are the variables that are linked by the branches at the smallest height. The closest relation was found between TMCA and Hex2Cer(d 18:1/26:0). The triacylglycerols TG (20:3_34:1) to TG (14:0_34:1) were relatively close to each other, while another group: IVA(NBS) to C3 of rather dissimilar variables is far from other variables. All TGs were quite similar to one another, with the closest relations among TG(18:1_33:0), TG(17:0_34:1), TG(18:1_33:1), TG(16:0_35:2), TG(16:0_33:1) and TG(17:1_34:1). Sum of purines appears as the most different from other variables, as it is linked at the largest height. Subsequent inherently unique variables are Sum of Betaine-Related Metabolites, Cystine Synthesis and Xanthine.

Significant Spearman rank correlations with the ranking points obtained by dogs in the competitions were identified for 3 variables: TG(16:0_35:2), TG(17:1_34:1) and TG(18:1_33:0) , all correlations were negative and only moderate (Supplementary Table S4).

## Discussion

This study is the first complex analysis of the changes in blood metabolic profile in the dogs subjected to regular sport training. The study design involved analyzing routine blood tests and metabolomic profile which gives much deeper insight into metabolic processes. The groups were selected to exclude the effect of the factors other than sport activity, mainly the diet, which could have affected the metabolism. ‘Biologically Approved Raw Food’ also called ‘Bone and Raw Food’ (BARF) is the most popular diet, favored by the owners and recommended for sports dogs. It generally consists of muscle meat, offal (organs), raw bone, fresh vegetables and fruit, and other healthy additives. It can be prepared at home or bought commercially. It is rich in saturated fats, so can affect metabolic parameters, i.e., concentrations of saturated triacylglycerols (triglycerides, TGs)^15^. That is why only dogs fed with homemade but unified BARF have been selected to ensure that the groups are as homogenous as possible, so the results can be understood as related to regular training. The food was prepared according to FEDIAF guidelines^16^ (as described in the Methods section). We have expected that similarly to humans and horses^17^ regular training produces changes related to energy metabolites at protein, carbohydrate and lipid levels and we wished to characterize them at metabolomic level and additionally, to check if any changes can be detected in the routine blood tests. The metabolomic analyses clearly indicated that training-related changes manifest mainly in the triacylglycerols profile and hardly in other profiles, and they are not pronounced in routine tests. We postulate that training-related metabolic shift in TGs profile improves the use of fatty acids as a source of energy during exertion.

Some significant differences between training and non-training dogs were revealed by routine hematological and blood biochemical tests, but the parameters remained within the reference ranges recommended by the laboratory in both groups, except for few individuals. Parameters describing red blood cells (RBC, HGB, HCT) did not differ significantly between training and non-training groups, and were rather in the upper limits of norms, which is a feature previously described and typical for Greyhounds and other sighthounds^13,18^. Thus, upper limits of ranges for canine species are recommended for these breeds but there is no need to use more specific values related to training.

Among blood biochemical parameters, creatine kinase (CK) is considered as the most important value related to training due to the fact that CK is the best indicator of striated muscle damage and most of its activity is in skeletal muscle^19,20^. The increase in plasma CK activity in dogs results from its leakage through the cell membrane and therefore, is evident in all conditions associated with muscle inflammation, necrosis, or degeneration but also after exhausting training^21^. Greyhounds have slightly higher CK activity than non-Greyhounds because of larger muscle mass^22^. In the dogs tested in this study, all CK results were within reference range and there was a significant difference between the training and non-training group in favour of the former. This is most likely due to regular training resulting in the increased muscle mass. However, FC_median_ was below 1.5, so this difference, although statistically significant, should not be ranked as important and meaningful.

Two biochemical parameters, lipase and gamma-glutamyltransferase activities, fulfilled both criteria FC_median_ above 1.5 and significant difference between training and non-training dogs. Lipase activity was almost 3 times higher in training dogs. In humans, it has been confirmed that the muscle lipoprotein lipase (LPL) increases significantly after exercise. This fact is related to the increased uptake of circulating triglycerides during exercise, which are either utilized immediately or used for restoration of muscle lipid stores after the end of exercise^23,24^. This is in line with our results indicating higher serum lipase levels in dogs that were in training compared to those that did not train actively. Although triglyceride concentrations in baseline biochemistry were not significantly different, metabolomic analysis clearly indicated the differences in TGs profiles between training and non-training dogs.

In case of GGT, high individual variations have been observed (from 5 to 22 U/L), however all of them were within the reference values. Serum GGT is derived from biliary epithelium, renal tubular epithelium, and pancreatic acinar cells^25^ and in the dogs truly elevated GGT levels together with elevated ALP indicate hepatic diseases but have not been discussed in the context of exercise. In humans, GGT increases acutely after exercise, but regular aerobic training has been shown to decrease GGT levels and the role of GGT in exercise has been linked to its role in glutathione metabolism, counteracting oxidative stress and supporting cells repair^26^. In racehorses, moderate increases in GGT levels, called ”GGT syndrome”, occur in some horses as a result of intensive training and is considered as a marker of maladaptation to training related to oxidative stress^27^. Due to the metabolic differences between horses and dogs it is hard to consider higher GGT level in training dogs as similar phenomenon, but this issue requires further research.

Total protein and albumin concentrations were lower in training dogs, which corresponds to lower total protein, albumin and globulin concentrations previously reported in Greyhounds and being suggested as related to plasma volume expansion associated with chronic conditioning and training^13^. Fructosamine is used to assess the average blood glucose concentration over the preceding 2–3 weeks. Although higher in training group, it remained within reference ranges, and reflected the temporary changes that may have had multiple reasons (including excitement related to training).

Metabolomic analysis clearly indicated that the training related metabolic changes involved primarily TGs’ levels. This class represented the majority (33 out of 48) of metabolites that significantly differed between groups, and the magnitude of change indicated by absolute FC_median_ above 1.5 confirmed their importance. They were also close to each other as indicated by hierarchical cluster analysis, with the closest relation among medium chain TGs with 16, 17 and 18 carbon atoms in *sn-1* position and 33, 34 and 35 carbon atoms in *sn-2* and *sn-3* positions. TGs are glycerides in which glycerol is esterified with 3 fatty acid groups. In the metabolomic data in our study they are described by the pattern TG(x:y_n:m) in which the number of carbon atoms (x) and double bonds (y) in the fatty acid residues at *sn-1* position and the total number of carbon atoms (n) and total number of double bonds (m) in the fatty acid residues at the positions *sn-2* and *sn-3* are given^28^.

Higher concentrations of TGs, primarily containing medium chain, having 16, 17 and 18 (with one double bond) carbon atoms at *sn-1* position (Fig. 3, Supplementary table S4) in training dogs seem to be related to the metabolic shift towards the increased availability of fatty acids as a source of energy used during training (repeated exercise).

During exercise, there are four major endogenous sources of energy: plasma glucose derived from liver glycogenolysis, free fatty acids (FFAs) released from adipose tissue lipolysis and from the hydrolysis of TG in very low-density lipoproteins (VLDL-TG), muscle glycogen and intramyocellular triacylglycerols (IMTGs) available within the skeletal muscle fibers. Endogenous TGs represent the largest energy reserve in the body, in humans it is over 60 times greater than the amount of energy stored in glycogen^24^. There are three pathways of TG biosynthesis, two of which take place in the liver and adipose tissue, and one in the intestines. Fats taken in with food are digested and undergo numerous transformations, requiring various enzymes, so as to be stored as cytoplasmic lipid droplets in all cell types of the body^29^. At rest FAs released from adipose tissue surpass the quantity of FAs oxidized in the skeletal muscle, most of the FAs are re-esterified into liver TGs^23^. When fatty acids are required by other tissues for energy or other purposes, they are released from the triacylglycerols by the sequential actions of three cytosolic enzymes^29^. The use of fatty acids as fuel requires hydrolysis of triacylglycerols (i.e., lipolysis) from adipose tissue, muscle, and plasma and the delivery of the released fatty acids to skeletal muscle mitochondria for oxidation^23^. Fat-based energy is twice as high (9 kcal/g, in the dogs 8.5 kcal/g) as the one based on protein or carbohydrates (4 kcal/g, in the dogs 3.5 kcal/g). Dogs even more than humans rely on free fatty acids for energy generation. In contrast to humans that use carbohydrate loading to increase stamina, in dogs exhaustion is not correlated to glycogen depletion. Dogs, during long lasting work at moderate intensity, continue to produce energy from fat oxidation^30,31^. Free fatty acids undergo beta oxidation to generate energy in the form of ATP in the post-absorption period. The length of the fatty acid chain plays the most important role in the amount of net energy gained, which is important during aerobic exercise. Medium- and short-chain fatty acids are more effectively oxidized, resulting in a higher thermogenic effect. On the other hand, long-chain fatty acids undergo oxidation less willingly and the amount of energy is lower than in the case of short- and medium-chain fatty acids. This is due, among other factors, to the need of activation of long-chain fatty acids before their oxidation, and the use of these compounds also as substrates for the formation of important biologically active molecules, such as eicosanoids. In addition, long-chain fatty acids, which constitute the majority of FFA derived from the diet or released from adipose tissue, unlike short- and medium-chain fatty acids, cannot pass directly through the mitochondrial membranes, but thanks to palmitoylcarnitine transferase 1 (CPT1) they are converted into fatty acylcarnitine derivatives^32^. The training-related metabolic changes detected in our study dealt with the composition of TGs in blood. In the dogs subjected to regular training, medium chain TGs predominated with the 16, 17 and 18 (with one double bond) carbon atoms residues in *sn-1* position, in contrast to non-training dogs, in which the residues containing 20 or 18 (but with 2 or 3 double bonds) carbon atoms predominated in this position. Thus, higher concentrations of TGs rich in medium chain fatty acids in the plasma of training dogs confirm that they are better available and preferentially used by dogs in the post-absorption period as the primary source of energy. The fact that different TGs predominated in the groups explains also that the concentration of TGs detected by routine blood biochemistry did not differ between training and non-training dogs. Unfortunately, only 3 TGs correlated with the ranking points and the correlations were only moderate. Thus, currently none of the tested metabolites could be proposed as an indicator of dog’s performance.

The interesting trend of TGs with 18 carbon atoms is worth mentioning. TG(18:0_30:1) to TG(18:1_38:7) were higher in the training group while TG(18:2_36:5) to TG(18:3_38:6) were lower. The changes in the concentrations of TGs with 18 carbon atoms have been reported in humans, however, the experimental design was different and involved the changes resulting from two handball games for 30 min with 10 min interval. It has been shown that the plasma levels of stearate (18:0) decreased, and oleate (18:1) and linoleate (18:2) increased, resulting in an increase of the ratio of unsaturated to saturated fatty acids^33^. This is in line with the findings in obese humans with impaired insulin sensitivity that had higher concentrations of stearic acid (18:0) and lower proportion of oleic (18:1) and linoleic (18:2) acids, giving a lower ratio of unsaturated to saturated fatty acids in skeletal muscle TGs—in comparison to non-obese individuals^34^. Mougios et al.^33^ assumed that in handball players the shift in blood TGs reflected the stimulation of releasing TGs from the liver during exercise which can overcome the hydrolysis of TG in muscles. They postulated, that the observed shift towards unsaturated fatty acids may be associated with aerobic exercise and diet rich in unsaturated fatty acids. Other study showed that in trained runners intramyocellular TGs provide a substantial portion of fatty acids fuel consumed during high intensity exercise, however there is still not enough data regarding the role of TG fatty acids to provide energy at rest or during exercise^35^.

We suspect that higher levels of some TGs in training dogs show their adaptation to intense running, as the source of needed energy is easily accessible from the blood. Still, the question that needs to be answered is: why some TGs concentrations are higher and some are lower when comparing trained to non-trained dogs? The obtained results give perspective and hope for determining the parameters that can be markers helping to set the training level of the organism and its adaptation to physical effort. However, further research is required to confirm this hypothesis. Metabolites belonging to other classes, representing other metabolic pathways, were only single among significantly different variables, which indicated that protein and carbohydrate metabolism were not markedly changed by the training. This fact is supported also by the relations among metabolites shown by hierarchical cluster analysis.

Interestingly, some metabolism indicators were significantly different between training and non-training groups with absolute FC_median_ above 1.5. Newborn screening (NBS) indicators reflect NBS traits mainly such as the deficiencies in enzymes that can produce certain inherited disorders in humans. This interpretation of metabolism indicators cannot be directly applied to dogs, but the differences between training and non-training dogs may suggest the role of genetic background related to the enzymes in the predisposition to the training. Training dogs usually originate from ”sport lines” which are selected by the owners interested in subjecting their dogs to sport training and competitions, but the breeding of ”sport lines” is based on the history of dogs and no specific genetic markers have been proposed. Our results may suggest that further research on the role of genetic predispositions may involve the genes related to certain enzymes.

The main limitation of this study is the small group of dogs, particularly the non-training ones. Moreover, training dogs took part in the competitions, which vary in terms of terrain and weather conditions, so the total exercise load was not the same for all dogs. BARF diet was prepared by the owner according to common recommendations; however, some minor differences may also have occurred. We did our best to select as homogenous groups as possible, but some differences certainly existed.

According to the authors’ knowledge, this is the first study covering a wide panel of metabolites in Whippet dogs, including the dogs in regular training. The results clearly indicated that in this homogenous group training produced changes mainly in TGs’ levels and other classes of metabolites were hardly changed. The detection of changes that seem aimed at more effective use of fatty acids as a source of energy during exertion can help in better understanding the possibilities of dietary interventions in dogs in regular training.

## Methods

Twenty-five adult Whippet dogs aged from 11 months to 5.5 years were enrolled in the study. The dogs were divided into two groups depending on whether they practiced lure coursing or not (Table 1) and these groups were compared. There were 16 dogs (14 males, including 4 neutered ones, and 2 females) that were in regular training and were competing in national and international coursing competitions. In addition to coursing training, consisting mainly of sprints in a straight line, each of the dogs practiced interval and endurance training with its owner, e.g., running by the bike, frisbee or flyball. During the sport events they competed against one another, regardless of their sex and age. The dogs were officially ranked due to the number of starts in the season and the places, so that better and worse competitors were indicated, 6 dogs were included into the ranking list but not ranked due to disqualifications and lack of enough competitions completed. The group of non-training dogs consisted of 9 dogs (4 males and 5 females) and apart from daily activities (e.g., walking, fetching toys) they did not practice any canine sport. All 25 dogs lived with their owners in domestic conditions. All dogs were fed with BARF (‘Biologically Approved Raw Food’ also called ‘Bone and Raw Food’) with addition of dietary supplements, such as vitamins and trace elements necessary for proper functioning of the body. When preparing meals for their dogs, the owners followed the recommendations of the guidelines of the European Pet Food Industry Federation (FEDIAF)^16^ and checked the designed diet with the same canine dietician. BARF diet consisted of 75% muscle meat, 5% offal (organs), 10% raw bone, 5% fresh vegetables and fruit, and 5% other healthy additives. The exact composition of the diet depended on the dogs’ and owners’ preferences but fulfilled FEDIAF criteria. As all dogs were considered adults, the amount of protein and fat based on MER (Maintenance Energy Requirements) of 110 kcal/kg was 45 g of protein and 13.75 g of fat (unit per 1000 kcal of metabolizable energy—ME). At the time of blood sampling, all dogs were clinically healthy, as confirmed by routine veterinary examination. The dogs were sampled at rest, all were fasted before blood sampling (8 h without food and 2 h without water). During the sampling the dogs were handled by their owners to minimize stress, as recommended by the Ethical Committee guidelines. Blood was collected indoors from either cephalic or saphenous vein using 0,8 mm needle into 1 ml plastic tubes coated with EDTA (for hematological analyses), heparin (for metabolomic analyses) and dry tubes coated with clotting factor (for blood biochemistry). Heparin tubes were centrifuged within 30 min after collection, at 2500 × *g* for 10 min, next the plasma was harvested and stored at − 80 °C.

All the procedures of blood sampling were performed as part of routine health examination, on the owners’ request and thus, according to the European directive EU/2010/63 and Polish regulations regarding experiments in animal^36^ there was no need for the approval of Ethical Committee for the described procedures, qualified as non-experimental clinical veterinary practices, excluded from the directive.

### Blood analyses

Within 4 h blood from EDTA tubes was tested using an automated hematology analyzer (Abacus, UK), in a laboratory for the following hematological measurements: white blood cells (WBC), red blood cells (RBC), hemoglobin (HGB), hematocrit (HCT), medium cell volume (MCV), medium cell hemoglobin (MCH), medium cell hemoglobin concentration (MCHC), red blood cell distribution width (RDW), platelets (PLT). The plain tubes were centrifuged, and the serum was used to evaluate biochemical profile consisting of the following parameters: alkaline phosphatase (ALP), glucose, creatinine, urea, total protein (TP), total bilirubin (TBIL), globulins (GLOB), albumins (ALB), gamma-glutamyltransferase (GGT), calcium (Ca), phosphorus (P), potassium (K), sodium (Na), magnesium (Mg), chloride (Cl), cholesterol, lactate dehydrogenase (LDH), creatine kinase (CK), triglycerides (TRI), amylase, lipase and fructosamine. For hematological and biochemical measurements reference intervals (RI) of the laboratory were used and compared to normal values for canine species^37^.

### Metabolomic analyses

Concentration of endogenous metabolites belonging to 23 classes (acylcarnitines, alkaloids, aminoacids, amino oxides, aminoacids related, bile acids, biogenic amines, carboxylic acids, ceramides, cholesterol esters, cresols, diacylglycerols, dihydroceramides fatty acids, glycerophospholipids, glycosylceramides, hormones, indoles and derivatives, nucleobases and related, sphingolipids, sugars, triacylglycerols, vitamins & cofactors) was determined by mass spectrometry using a commercial MetaboINDICATOR™: MxP® Quant 500 Kit (Biocrates Life Sciences AG, Innsbruck, Austria). The kit is designed to measure concentration of 630 metabolites: 40 acylcarnitines, 1 alkaloid, 1 amine oxides, 20 aminoacids, 30 aminoacids related, 14 bile acids, 9 biogenic amines, 7 carboxylic acids, 28 ceramides, 22 cholesterol esters, 1 cresol, 44 diacylglycerols, 8 dihydroceramides, 12 fatty acids, 90 glycerophospholipids, 34 glycosylceramides, 4 hormones, 4 indoles and derivatives, 2 nucleobases and related, 15 sphingolipids, 1 sugars, 242 triacylglycerols, 1 vitamin & cofactors. Moreover, 232 metabolism indicators, meaning predefined biologically metabolite sums and ratios, were measured.

Briefly, a 96-well based sample preparation device was used to quantitatively analyze the metabolite profile in the samples. This device consisted of inserts that had been impregnated with internal standards, and a predefined sample amount was added to the inserts. Next, a phenyl isothiocyanate (PITC) solution was added to derive some of the analytes, and after the derivatization had been completed, the target analytes were extracted using an organic solvent, followed by a dilution step. The obtained extracts then were analyzed by LC–MS/MS (small molecules, bile acids, and free fatty acids) and FIA-MS/MS (lipid classes including acylcarnitines and hexoses) methods, using multiple reaction monitoring to detect the analytes. Data items were quantified using appropriate mass spectrometry software (Sciex Analyst) and imported into Biocrates. MetIDQ software (Biocrates Life Sciences AG, Innsbruck, Austria) for further analysis^15^. The analysis was run in Fraunhofer-Institute for Toxikology and Experimental Medicine ITEM in Hannover, Germany, in March 2022. Concentrations of all metabolites were calculated in μM (μmol/L) and were normalized with respect to internal quality control samples. Individual limit of detection (LOD) was calculated for each metabolite. In total, concentration of 630 metabolites was measured, of which 157 results were rejected due to being beyond LOD.

### Statistical analysis

The significance of the differences between the concentrations of metabolites in training and non-training groups was checked by Mann–Whitney U test. The same test was applied to evaluate the significance of the differences in hematological and blood biochemistry parameters between the examined groups. *P* < 0.05 was considered as significant.

To evaluate possible importance of the differences in metabolites concentrations between training and non-training dogs fold change (FC_median_) based on medians was calculated according to previously described formula:

$${\text{FCmedian}} = {\text{Me1}}/{\text{Me}}0\;{\text{if}}\;{\text{Me1}} > {\text{Me}}0\;{\text{or}}\;{\text{FCmedian}} = - {\text{Me}}0/{\text{Me1}}\;{\text{if}}\;{\text{Me1}} < {\text{Me}}0$$

where Me_0_ and Me_1_ are medians for training and non-training groups, respectively^38^. The values higher then absolute cut-off 1.5 were treated as possibly important.

To investigate the relations among the metabolites and metabolism indicators, the variables that differed significantly between groups and the absolute FC_median_ which exceeded 1.5 were standardized and subjected to the single linkage hierarchical clustering with Euclidean distance.

To evaluate the relations between ranking points and metabolites concentrations Spearman R correlations were calculated. Data are presented as the median (Me), interquartile range (IQR) and range. Statistica 13.3.0 (TIBCO) was used for the calculations.

### Supplementary Information

Supplementary Tables.

Supplementary Information 2.

Supplementary Tables.

## Data Availability

The datasets generated during and/or analyzed during the current study are available from the corresponding author on reasonable request.

## References

[1] Lv Z, Gong ZG, Xu YJ (2022). Research in the field of exercise and metabolomics: A bibliometric and visual analysis. Metabolites.

[2] Belhaj MR, Lawler NG, Hoffman NJ (2021). Metabolomics and lipidomics: Expanding the molecular landscape of exercise biology. Metabolites.

[3] Krasztel MM (2022). Correlation between metabolomic profile constituents and feline pancreatic lipase immunoreactivity. J. Vet. Intern. Med..

[4] Muñoz-Prieto A (2021). Evaluation of changes in metabolites of saliva in canine obesity using a targeted metabolomic approach. Animals.

[5] Carlos G, Paulo dos Santos F, Fröehlich PE (2022). Canine metabolomics advances. Metabolomics.

[6] Milczarek M (2019). Metabolomic profile of adult Saanen goats infected with small ruminant lentovirus. Small Rumin. Res..

[7] Kelly RS, Kelly MP, Kelly P (2020). Metabolomics, physical activity, exercise and health: A review of the current evidence. Biochim. Biophys. Acta Mol. Basis Dis..

[8] Bongiovanni T (2019). Sportomics: Metabolomics applied to sports. The new revolution?. Eur. Rev. Med. Pharmacol. Sci..

[9] Duft RG, Castro A, Chacon-Mikahil MPT, Cavaglieri CR (2017). Metabolomics and exercise: Possibilities and perspectives. Motriz Rev. Educ. Fis..

[10] Morville T, Sahl RE, Moritz T, Helge JW, Clemmensen C (2020). Plasma metabolome profiling of resistance exercise and endurance exercise in humans. Cell Rep..

[11] Cai M (2022). Blood metabolomics analysis identifies differential serum metabolites in elite and sub-elite swimmers. Front. Physiol..

[12] Pellegrino FJ, Risso A, Vaquero PG, Corrada YA (2018). Physiological parameter values in greyhounds before and after high-intenisty exercise. Open Vet J..

[13] Zaldívar-López S (2011). Clinical pathology of greyhounds and other sighthounds. Vet. Clin. Pathol..

[14] Miazga K (2023). Exercise-induced haematological and blood lactate changes in Whippets training for lure coursing. J. Vet. Res..

[15] Alexopolus AS (2019). Triacylglycerides: Emerging targets in diabetes care? Review of moderate hipetriglycerydemia in diabetes. Curr. Diab. Rep..

[16] FEDIAF Nutritional Guidelines for Complete and Complementary Pet Food for Cats and Dogs, *European Pet Food* https://europeanpetfood.org/wpcontent/uploads/2022/03/Updated-Nutritional-Guidelines.pdf (2021).

[17] Ohmura H (2021). Metabolomic analysis of skeletal muscle before and after strenuous exercise to fatigue. Sci. Rep..

[18] Uhrikova I (2013). Haematological and biochemical variations among eight sighthound breeds. Aust. Vet. J..

[19] Baird MF, Graham SM, Baker JS, Bickerstaff GF (2012). Creatine kinase and exercise-related muscle damage implications for muscle performance and recovery. J. Nutr. Metab..

[20] Koch AJ, Pereira R, Machado M (2014). The creatine kinase response to resistance exercise. J. Musculosckelet. Neuronal. Interact..

[21] Spinella G (2021). Clinical evaluation of creatine kinase and aspartate aminotransferase for monitoring muscle effort in working dogs in different simulated fieldworks. Animals.

[22] Dunlop MM (2011). Determination of serum biochemistry reference intervals in a large sample of aduls greyhounds. J. Small Anim. Pract..

[23] Horowitz JF, Klein S (2000). Lipid metabolism during endurance exercise. Am. J. Clin. Nutr..

[24] Muscella A, Stefáno E, Lunetti P, Capobianco L, Marsigliante S (2021). The regulation of fat metabolism during aerobic exercise. Biomolecules.

[25] Brennan PN, Dillon JF, Tapper EB (2021). Gamma-glutamyl transferase – and old dog with new tricks?. Liver Int..

[26] Fragala MS (2017). Associations of aerobic and strength exercise with clinical laboratory test values. Plos One.

[27] Mann S (2022). Investigating the pathogenesis of high-serum gamma-glutamyl transferase activity in Thoroughbred racehorses: A series of case-control studies. Equine Vet. J..

[28] Czopowicz M (2020). Profile of serum lipid metabolites of one-week-old goat kids depending on the type of rearing. BMC Vet. Res..

[29] Christie, W. W. Triacylglycerols: 2. Biosythesis and Metabolism. *The Lipid Web* https://www.lipidmaps.org/resources/lipidweb/lipidweb_html/lipids/simple/tag2/index.htm (2023).

[30] Zoran DL (2021). Nutrition of working dogs: Optimal performance and health. Vet. Clin. North. Am. Small Anim..

[31] Wakshlag J, Shmalberg J (2014). Nutrition for working and service dogs. Vet. Clin. North. Am. Small Anim..

[32] Meienberg F (2019). The effect of exercise on intramyocellular acetylcarnitine (AcCtn) concentration in adult growth hormone deficiency (GHD). Sci. Rep..

[33] Mougios V, Kotzamanidis C, Koutsari C, Atsopardis S (1995). Exercise-induced changes in the concentration of individual fatty acids and triacyloglycerols in human plasma. Metabolism.

[34] Manco M (2000). Insulin resistance directly correlates with increased saturated fatty acids in skeletal muscle triglycerides. Metabolism.

[35] Jensen MD (2003). Fate of fatty acids at rest and during exercise: regulatory mechanisms. Acta Physiol. Scand..

[36] Act of 15 January 2015 on the protection of animals used for scientific or educational purposes, art 1.2 (5), Dz.U.2018.0.1207.

[37] Winnicka A. *Reference values of basic laboratory measurements in veterinary* [in Polish], 108, (SGGW, 2021).

[38] Vinaixa M (2015). Mass spectral datababses for LC/MS- and GC/MS-based metabolomics: State of the field and future prospects. Trac-trend Anal. Chem..

