# Supplementary material for: Metabolomic analysis indicated changes in triacylglycerols’ levels as a result of training in Whippet dogs
Source: Sci Rep. 2023 Oct 25;13:18223. doi: 10.1038/s41598-023-45546-w (PMC10600122; doi:10.1038/s41598-023-45546-w)
Supplement: Supplementary file 1 — Supplementary Tables. [file 41598_2023_45546_MOESM1_ESM.docx]

Supplementary Table S1. Additional hematology results of training and not training Whippets at rest

| **Blood parameter** | **Reference interval** | **Training** | | **Non-traning** | | **Training**  **vs non-training**  **p-value*** | **FC** |
| --- | --- | --- | --- | --- | --- | --- | --- |
|  |  | **Median (IQR)** | **Range** | **Median (IQR)** | **Range** |  |  |
| **MCV**  **[fl]** | **60-77** | 71.5 (70.75-74) | (69-75) | 73.2 (72-74.3) | (69.8-77) | 0.21 | 1.024 |
| **MCH**  **[fmol]** | **1.18-1.55** | 1.53 (1.51-1.57) | (1.47-1.61) | 1.59 (1.56-1.6) | (1.46-1.62) | 0.12 | 1.043 |
| **MCHC**  **[mmol/l]** | **19.8-22.3** | 21.45 (21.18-21.6) | (20.02-21.9) | 21.5 (21.3-21.6) | (20.8-22) | 0.67 | 1.002 |
| **PLT**  **[G/l]** | **200-580** | 216.5 (200-226) | (177-245) | 208 (181-215) | (178-241) | 0.39 | -1.041 |

* Significant at α=0.05

MCV – medium cell volume, MCH – medium cell hemoglobin, MCHC –medium cell hemoglobin concentration, RDW -red blood cell distribution width, PLT – platelets

Supplementary Table S2. Additional blood biochemistry results of training and not training Whippets at rest

| **Blood parameter** | **Reference interval** | **Training** | | **Non-training** | | **Training**  **vs non- training**  **p-value** | **FC** |
| --- | --- | --- | --- | --- | --- | --- | --- |
|  |  | **Median (IQR)** | **Range** | **Median (IQR)** | **Range** |  |  |
| **ALP**  **[U/l]** | **5 – 155** | 56 (18-85.5) | (14-122) | 28 (14-32) | (11-40) | 0.11 | -2 |
| **Glucose**  **[mg/dl]** | **70 – 120** | 84 (74-98) | (56-112) | 116 (108-116) | (102-128) | <0.00 | 1.381 |
| **Creatinine**  **[mg/dl]** | **0.8 – 1.7** | 1 (0.9-1.1) | (0.8 – 1.3) | 0.8 (0.8-1) | (0.8-1) | 0.122 | -1.250 |
| **Urea**  **[mg/dl]** | **20 – 50** | 35 (27.5-43.5) | (22 – 55) | 35 (31-41) | (26-50) | 0.86 | 1 |
|  |  |  |  |  |  |  |  |
| **TBIL**  **[mg/dl]** | **0.3 – 0.9** | 0.3 (0.3-0.5) | (0.3 – 0.6) | 0.4 (0.3-0.4) | (0.3-0.4) | 0.636 | 1.333 |
| **GLOB**  **[g/l]** | **28 – 42** | 24 (23-25) | (20 – 28) | 24 (22.25-25.75) | (20-27) | 0.696 | 1 |
|  |  |  |  |  |  |  |  |
| **Ca**  **[mg/dl]** | **8.4 – 11.5** | 9.9 (9.5-10) | (9.0 – 10.6) | 10.7 (10.45-10.8) | (9.2-10.8) | 0.023 | 1.081 |
| **P**  **[mmol/l]** | **2.5 – 6.3** | 3.2 (3-3.8) | (2.6 – 5.4) | 2.85 (2.58-2.98) | (2.4-3.9) | 0.066 | -1.123 |
| **K**  **[mmol/l]** | **4.1 – 5.4** | 4.22 (4.1-4.39) | (4.0 – 5.1) | 5.21 (4.74-5.31) | (4.09-5.35) | 0.079 | 1.214 |
| **Na**  **[mmol/l]** | **139.1 – 156.5** | 149.25 (148.43-151.5) | (143.4 – 162.3) | 156 (153.53-157) | (148.5-157.6) | 0.192 | 1.054 |
| **Mg**  **[mg/dl]** | **1.7 – 2.9** | 1.9 (1.8-1.9) | (1.7 – 2.1) | 1.8 (1.73-1.95) | (1.7-2.1) | 0.444 | -1.056 |
| **Cl**  **[mmol/l]** | **98.7 – 120.6** | 112.92 ±3.56 | (108.8 – 120.2) | 114.13 ± 1.98 | (112.2-116.6)) | 0.441 | 1.015 |
| **Cholesterol**  **[mg/dl]** | **127.7 – 360** | 156.5 (135.5-176.25) | (106 – 307) | 181 (152-236) | (152-236) | 0.256 | 1.157 |
|  |  |  |  |  |  |  |  |

* Significant at α=0.05

ALP – alkaline phosphatase, TBIL – total bilirubin, GLOB – globulins, Ca – calcium, P- phosphorus, K – kalium, Na – sodium, Mg – magnesium, Cl – chloride, , CK – creatine kinase,
