# Supplementary material for: Metabolomic analysis indicated changes in triacylglycerols’ levels as a result of training in Whippet dogs
Source: Sci Rep. 2023 Oct 25;13:18223. doi: 10.1038/s41598-023-45546-w (PMC10600122; doi:10.1038/s41598-023-45546-w)
Supplement: Supplementary file 3 — Supplementary Tables. [file 41598_2023_45546_MOESM3_ESM.docx]

Supplementary Table S4. Concentrations of metabolites that significantly (p<0.05) differed between training and non-training dogs. r is given for the metabolites significantly correlated with the ranking points. NS – not significant

| **Metabolite** | **Training** | | **Non training** | | **FC_median_^a^** | **r** | **p** |  |
| --- | --- | --- | --- | --- | --- | --- | --- | --- |
|  | **Median (IQR)** | **Range** | **Median (IQR)** | **Range** |  |  |  |  |
| **Free fatty acids** | | | | | | | | |
| FA(18:2) | 28.25 (17.775- 36.125) | 0 - 67 | 52.4 (34.8 – 62.6) | 0.64 – 88.6 | -1.855 | NS | 0.004 |  |
| FA(20:1) | 1.055 (0 – 2.84) | 0 -6.62 | 5.59 (3.24 – 6.67) | 0.849 – 12 | -5.298 | NS | 0.001 |  |
| FA(20:3) | 0.622 (0.551 – 0.699) | 0-1.39 | 1.22 (0.789 – 1.5) | 0.739 – 2.49 | -1.961 | NS | 0.001 |  |
| **Acylcarnitines** | | | | | | | | |
| C3 | 0.196 (0.174-0.253) | 0.095 – 0.325 | 0.121 (0.104 – 0.163) | \| 0.101 – 0.22 \| \| --- \| \|  \| | 1.62 | NS | 0.017 |  |
| **Aminoacids Related** | | | | | | | | |
| Kyneurine | 4.415 (3.46 – 6.155) | 1.71-8.08 | 2.22 (1.88-3.23) | 0.626 – 7.6 | 1.989 | NS | 0.023 |  |
| **Bile Acids** | | | | | | | | |
| TMCA | 0.108 (0.056- 0.259) | 0.016 – 0.574 | 0.028 (0.019-0.055) | 0.004 – 25.9 | 3.857 | NS | 0.02 |  |
| **Biogenic Amines** | | | | | | | | |
| Serotonin | 0.18 (0.126 – 0.223) | 0 – 0.682 | 0.093 (0 – 0.191) | 0 – 0.834 | 1.935 | NS | 0.027 |  |
| **Carboxylic Acids** | | | | | | | | |
| Suc | 19.65 (17.35 – 23.275) | 0 – 29.9 | 12.1 (10.2 – 12.9) | 0 – 14.8 | 1.624 | NS | <0.001 |  |
| **Cholesterol Esters** | | | | | | | | |
| CE (17:0) | 11.85 (8.515 – 14.475) | 5.59 – 18.5) | 6.46 (5.46 – 8.81) | 0.043 – 16.4 | 1.834 | NS | 0.043 |  |
| CE (22:6) | 10.85 (4.548 – 17.875) | 1.86 – 41.8 | 21.4 (13.3 – 33.6) | 0.517 – 39.3 | -1.972 | NS | 0.027 |  |
| **Cresols** | | | | | | | | |
| p-Cresol-SO4 | 0.75 ( 0.575 – 1.315) | 0.142 – 2.96 | 0.392 (0.175 – 0.526) | 0.097 – 0.829 | 1.995 | NS | 0.01 |  |
| **Glycerophospholipids** | | | | | | | | |
| PC aa C38:0 | 3.095 (2.303 – 4.845) | 1.43 – 7.1 | 5.7 (4.99 – 6.13) | 2.95 – 9.98 | -1.842 | NS | 0.023 |  |
| PC aa C40:6 | 29.95 (15.625 – 52.475) | 9.97 – 74.1) | 57.5 (33 – 62.2) | 0.133 – 93.5 | -1.92 | NS | 0.032 |  |
| **Glycosylceramides** | | | | | | | |  |
| HexCer (d18:1/26:0) | 0.004 (0-0.004) | 0 – 0.009 | 0.006 (0.004 – 0.008) | 0 - 189 | -1.5 | NS | 0.043 |  |
| **Nucleobases Related** | | | | | | | | |
| Xanthine | 0.361 (0.23 – 0.424) | 0 – 0.695 | 0.6 (0.562 – 0.715) | 0.334 – 0.846 | -1.662 | NS | 0.001 |  |
| **Triacylgycerols** | | | | | | | |  |
| TG(14:0_34:1) | 2.79 (1.533 – 3.593 | 0.808 – 5.47 | 1.63 (1.19 – 1.74) | 0.285 – 2.97) | 1.712 | NS | 0.037 |  |
| TG(14:0_39:3) | 0.018 (0.015 – 0.027) | 0 – 0.043 | 0.033 (0.03 – 0.038) | 0 – 0.285 | -1.833 | NS | 0.032 |  |
| TG(16:0_32:0) | 4.29 (2.62-5.813) | 2.15 – 10.6 | 2.63 (2.12-4.14) | 0.134 – 4.46 | 1.631 | NS | 0.027 |  |
| TG(16:0_32:1) | 5.55 (4.195 – 7.235) | 1.69 – 10.3 | 3.29 (3.04 – 4.09) | 1.98 – 5.24 | 1.686 | NS | 0.014 |  |
| TG(16:0_33:1) | 1.905 (1.088 – 2.648) | 0.737 – 2.96 | 0.972 (0.645 – 1.13) | 0.612 – 1.27 | 1.96 | NS | 0.002 |  |
| TG(16:0_35:1) | 2.24 (1.348 – 2.935) | 0.818 – 3.66 | 1.2 (0.799 – 1.4) | 0.532 – 5.53 | 1.867 | NS | 0.003 |  |
| TG(16:0_35:2) | 1.91 (1.215 – 2.218) | 0.891 – 2.79 | 1.11 (0.779 -1.3) | 0.662 – 1.48 | 1.721 | -0.53 | 0.004 |  |
| TG(17:0_32:1) | 0.435 (0.346 – 0.648) | 0.239 – 0.819 | 0.283 (0.248-0.309 | 0 – 0.384 | 1.539 | NS | 0.002 |  |
| TG(17:0_34:1) | 1.96 (1.285 – 2.918) | 0.746 – 3.57 | 0.882 (0.599 – 1.39) | 0.586 – 1.46 | 2.222 | NS | 0.005 |  |
| TG(17:1_34:1) | 1.465 (0.938 – 1.825) | 0.483 – 2.36 | 0.832 (0.603 – 0.981) | 0.259 – 1.08 | 1.761 | -0.55 | 0.01 |  |
| TG(18:0_30:1) | 0.37 (0.244 – 0.578) | 0.151 – 0.817 | 0.17 (0.153 – 0.3) | 0 – 0.471 | 2.179 | NS | 0.043 |  |
| TG(18:1_30:0) | 3.675 (2.533 – 4.8) | 1.12 – 6.24 | 1.99 (1.44 -2.44) | 0.699 -3.24 | 1.847 | NS | 0.008 |  |
| TG(18:1_30:1) | 1.48 (1.153 – 2.185) | 0.584 – 6.5 | 0.846 (0.546 – 1.17) | 0.39 – 1.86 | 1.749 | NS | 0.043 |  |
| TG(18:1_33:0) | 2.555 (1.385 – 3.29) | 0.943 – 4.84 | 1.15 (0.715 – 1.53) | 0.53 – 1.62 | 2.222 | -0.51 | 0.004 |  |
| TG(18:1_33:1) | 3.7 (2.558 – 4.498) | 1.49 – 7.2 | 1.49 (1.02 – 2.28) | 0.561 – 2.8 | 2.483 | NS | 0.001 |  |
| TG(18:1_34:1) | 68.05 (51.7 – 87.6) | 28.6 - 146 | 44.5 (37.9 – 56.1) | 0.092 - 101 | 1.529 | NS | 0.027 |  |
| TG(18:1_35:2) | 2.6 (2.305 – 3.038) | 1.13 – 4.5 | 1.17 (1.03 – 1.73) | 0.011 – 2.66 | 2.222 | NS | 0.001 |  |
| TG(18:1_36:1) | 15.65 (11.6 – 22.25) | 7.04 – 44.7 | 10.2 (8.03 – 12.4) | 0.095 – 21.5 | 1.534 | NS | 0.023 |  |
| TG(18:1_36:2) | 33.8 (25.425-40.525) | 13.5 – 81.8 | 21 (17.3 – 30.1) | 0.18 - 48 | 1.610 | NS | 0.027 |  |
| TG(18:1_38:7) | 0.759 (0.568 – 1.033) | 0.33 – 1.56 | 1.26 (1.08-1.36) | 0.823 – 6.33 | -1.66 | NS | 0.008 |  |
| TG(18:2_35:1) | 1.74 (1.265 – 2.08) | 1.07 – 2.68 | 1.12 (0.82 – 1.33) | 0.538 – 2.14 | 1.554 | NS | 0.014 |  |
| TG(18:2_36:5) | 0.65 (0.533 – 0.962) | 0.154 – 2.18 | 1.22 (1.09 – 1.39) | 0.637 – 5.11 | -1.876 | NS | 0.012 |  |
| TG(18:2_38:5) | 2.14 (1.243 – 2.603) | 0.703- 5.35 | 3.23 (2.67 – 3.49) | 2-3.61 | -1.509 | NS | 0.014 |  |
| TG(18:2_38:6) | 1.085 (0.503 – 1.423) | 0.198 – 2.96 | 1.89 (1.37 – 1.91) | 0.257 – 2.64 | -1.742 | NS | 0.017 |  |
| TG(18:3_38:5) | 0.404 (0.277 – 0.634) | 0.111 – 1.32 | 0.71 (0.552 – 0.924) | 0.25 – 1.12 | -1.757 | NS | 0.012 |  |
| TG(18:3_38:6) | 0.299 (0.189 – 0.419) | 0 – 1.29 | 0.693 (0.555-0.754) | 0.125 – 1.25 | -2.317 | NS | 0.005 |  |
| TG(20:3_32:0) | 0.64 (0.586 – 0.95) | 0.287 – 1.14 | 0.357 (0.339 – 0.492) | 0.191 – 0.686 | 1.793 | NS | 0.004 |  |
| TG(20:4_32:2) | 0.5 (0.427 – 0.893) | 0.196 – 1.45 | 0.847 (0.2 – 0.706) | 0.571 – 1.09 | -1.694 | NS | 0.017 |  |
| TG(20:4_34:2) | 5.025 (3.293 – 6.898) | 2.03 – 16.2 | 8.03 (7.61 – 10.1) | 0.365 – 15.9 | -1.588 | NS | 0.01 |  |
| TG(20:4_34:3) | 0.975 (0.708 – 1.82) | 0.303 – 3.74 | 2.54 (2.03 – 2.91) | 0.935 – 17.2 | -2.606 | NS | 0.012 |  |
| TG(20:4_36:3) | 5.78 (2.995 – 7.185) | 1.81 – 18.2 | 10.2 (8.27 – 10.9) | 5.45 – 15.1 | -1.765 | NS | 0.012 |  |
| TG(20:4_36:4) | 2.28 (1.58 – 4.24) | 1.18 – 9.98 | 5.45 (5.25 – 6.46) | 2.56 – 7.37 | -2.39 | NS | 0.007 |  |
| TG(20:4_36:5) | 0.825 (0.581 – 1.608) | 0.231 – 4.07 | 2.27 (1.55 – 3.27) | 1.05 – 4.43 | -3.03 | NS | 0.003 |  |

Supplementary Table S5. Concentrations of metabolite indicators that significantly (p<0.05) differed between training and non-training dogs. None of the metabolism indicators significantly correlated with the ranking points (NS – not significant)

| **Metabolite indicator** | **Training** | | **Non training** | | **FC_median_** | **r** | **p** |
| --- | --- | --- | --- | --- | --- | --- | --- |
|  | **Median (IQR)** | **Range** | **Median (IQR)** | **Range** |  |  |  |
| IBD Deficiency (NBS) | 0.029 (0.023 – 0.033) | 0.018 – 0.053 | 0.018 (0.016-0.027) | 0.015 – 0.035 | 1.583 | NS | 0.027 |
| IVA (NBS) | 0.078 (0.047-0.095) | 0.026 – 0.156 | 0.031 (0.029-0.038) | 0.024-0.06 | 2.5 | NS | 0.001 |
| MA (NBS) | 0.108 (0.066-0.145) | 0.029- 0.192 | 0.051 (0.036-0.056) | \| 0.025 – 0.076 \| \| --- \| | 2.118 | NS | <0.001 |
| MC Deficiency (NBS) | 0.65 (0.616-0.799) | 0.374 – 1.74 | 1.230 (0.972- 1.32) | 0.802 – 2.52 | -1.892 | NS | <0.001 |
| MMA (NBS) | 0.008 (0.005-0.009) | 0.003 – 0.01 | 0.004 (0.003-0.005) | 0.003 – 0.009 | 1.875 | NS | 0.01 |
| PA (NBS) | 1.54 (1.250 (1.625) | 0.575 – 2.67 | 0.815 (0.775 – 1.03) | 0.396 – 1.25 | 1.89 | NS | 0.001 |
| SCAD Deficiency (NBS) | 0.275 (0.245-0.341) | 0.156 – 0.623 | 0.489 (0.319-0.557) | 0.269 – 0.592 | -1.778 | NS | 0.014 |
| Sum of Betaine-Relates Metabolites | 0.315 (10.285-0.469) | 0.149 – 0.655 | 0.57 (0.407 – 0.745) | 0.271 – 1.63 | -1.81 | NS | 0.017 |
| Cystine Synthesis | 0.381 (0.343 – 0.453) | 0.267 – 0.578 | 0.711 (0.414-0.831) | 0.326 – 0.955 | -1.866 | NS | 0.014 |
| p-cresol-SO4 Synthesis | 0.014 (0.011-0.023) | 0.003 – 0.061 | 0.007 (0.004-0.011) | 0.002 – 0.016 | 2 | NS | 0.023 |
| Sum of VLCFA-CEs | 14.25 (6.92 – 20.225) | 3.28 – 49.8 | 25.7 (17.5 – 36.6) | 10.4 – 43.2 | -1.804 | NS | 0.023 |
| Ratio of DGs to TGs | 0.006 (0.005 – 0.007) | 0.002 – 0.012 | 0.009 (0.008-0.011) | 0.008 – 0.012 | -1.636 | NS | 0.004 |
| Ratio of DHA to EPA | 3.59 (2.503 – 4.395) | 1.66 – 7.29 | 5.4 (4.55 – 6.18) | 3.6 – 7.01 | -1.504 | NS | 0.023 |
| Sum of MUFAs | 232.5 (177-279.75) | 96.7 - 561 | 326 (298-505) | 136 - 530 | -1.402 | NS | 0.037 |
| Sum of PUFAs | 39.15 (26.85 -58.725) | 6.29 – 99.3 | 79 (37.257 – 104) | 40.6 - 151 | -2.018 | NS | 0.004 |
| PLA2 Activity (3) | 0.366 (0.324 – 0.581) | 0.142 – 1.16 | 0.602 (0.53-0.935) | 0.375 – 1.22 | -1.647 | NS | 0.017 |
| Ratio of TGs to FAs | 2.265 (1.863-3.31) | 1.38 – 6.23 | 1.35 (0.804 – 2.71) | 0.494 – 3.16 | 1.678 | NS | 0.043 |
| Sum of Purines | 0.414 (0.24-0.71) | 0.186 – 2.4 | 1.57 (1.29-1.87) | 0.944 – 2.55 | -3.792 | NS | <0.001 |
| Sum of Saturated TGs | 13.05 (8.315-17.825) | 5.45 – 34.2 | 8.24 (7.37 – 10.3) | 3.21 – 14.9 | 1.584 | NS | 0.049 |
